# Meeting Report of the Second Symposium of the Belgian Society for Viruses of Microbes and Launch of the Phage Valley

**DOI:** 10.3390/v16020299

**Published:** 2024-02-15

**Authors:** Salomé Desmecht, Agnieszka Latka, Pieter-Jan Ceyssens, Abel Garcia-Pino, Annika Gillis, Rob Lavigne, Gipsi Lima-Mendez, Jelle Matthijnssens, Roberto Vázquez, Jolien Venneman, Jeroen Wagemans, Yves Briers, Damien Thiry

**Affiliations:** 1 Veterinary Bacteriology, Department of Infectious and Parasitic Diseases, Fundamental and Applied Research for Animals and Health, Faculty of Veterinary Medicine, University of Liège (ULiège), 4000 Liège, Belgium; salome.desmecht@uliege.be; 2 Laboratory of Applied Biotechnology, Department of Biotechnology, Faculty of Bioscience Engineering, University of Ghent (UGent), 9000 Gent, Belgium; agnieszka.latka@ugent.be (A.L.); roberto.vazquezfernandez@ugent.be (R.V.); 3 Department of Pathogen Biology and Immunology, Faculty of Biological Sciences, University of Wroclaw, 51-148 Wroclaw, Poland; 4 Division of Human Bacterial Diseases, Sciensano, 1050 Brussels, Belgium; pieter-jan.ceyssens@sciensano.be; 5 Cellular and Molecular Microbiology, Faculty of Sciences, Université Libre de Bruxelles (ULB), 1050 Brussels, Belgium; abel.garcia.pino@ulb.be; 6 Laboratory of Food and Environmental Microbiology, Earth and Life Institute, Catholic University of Louvain (UCLouvain), 1348 Louvain-la-Neuve, Belgium; annika.gillis@uclouvain.be; 7 Laboratory of Gene Technology, Department of Biosystems, Faculty of Bioscience Engineering, KU Leuven, 3001 Leuven, Belgium; rob.lavigne@kuleuven.be (R.L.); jeroen.wagemans@kuleuven.be (J.W.); 8 Biology of Microorganisms Research Unit (URBM), Namur Research Institute for Life Sciences (NARILIS), University of Namur (UNamur), 5000 Namur, Belgium; gipsi.limamendez@unamur.be; 9 Laboratory of Viral Metagenomics, Department of Microbiology, Immunology and Transplantation, Rega Institute, Division of Clinical and Epidemiological Virology, KU Leuven, 3000 Leuven, Belgium; jelle.matthijnssens@kuleuven.be; 10 Flanders Research Institute for Agriculture, Fisheries and Food (ILVO), 9820 Merelbeke, Belgium; jolien.venneman@ilvo.vlaanderen.be

**Keywords:** Belgian Society for Viruses of Microbes, bacteriophages, triple helix, phage therapy, Phage Valley

## Abstract

The second symposium of the Belgian Society for Viruses of Microbes (BSVoM) took place on 8 September 2023 at the University of Liège with 141 participants from 10 countries. The meeting program covered three thematic sessions opened by international keynote speakers: two sessions were devoted to “Fundamental research in phage ecology and biology” and the third one to the “Present and future applications of phages”. During this one day symposium, four invited keynote lectures, nine selected talks and eight student pitches were given along with thirty presented posters. The president of the Belgian Society for Viruses of Microbes, Prof. Yves Briers, took advantage of this symposium to launch the Phage Valley concept that will put the spotlight on the exceptionally high density of researchers investigating viruses of microbes as well as the successful triple helix approach between academia, industry and government in Belgium.

## 1. Introduction

**Figure 1:**
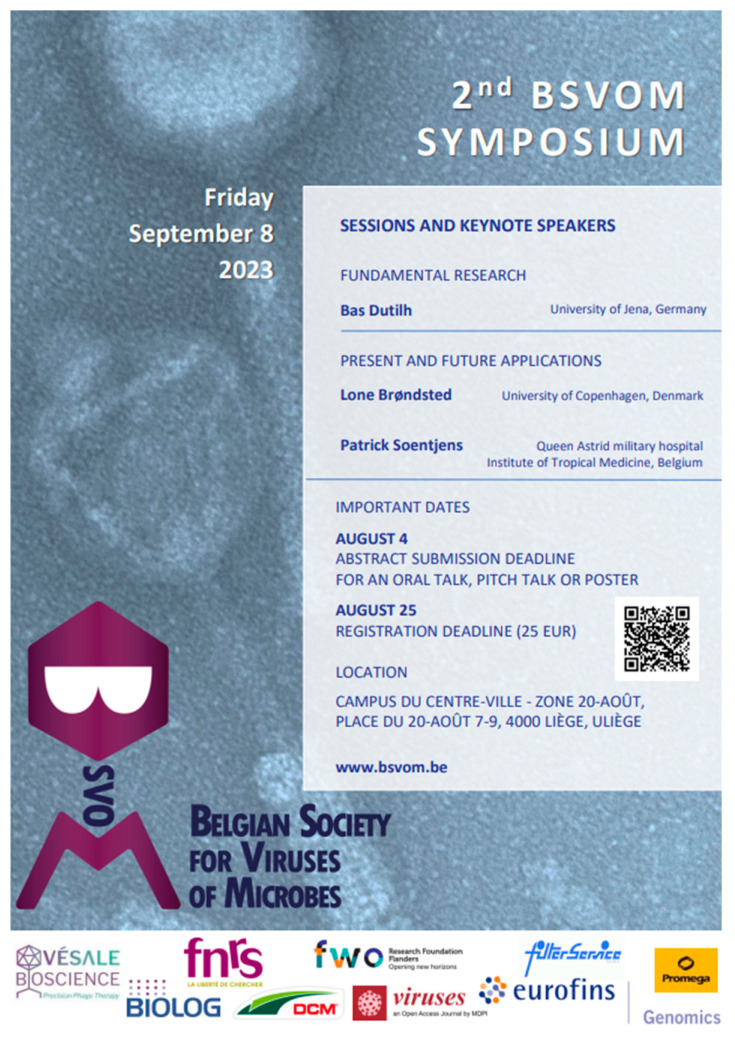
Flyer advertising the 2nd Belgian Society for Viruses of Microbes (BSVoM) symposium (with sponsors).

**Figure 2:**
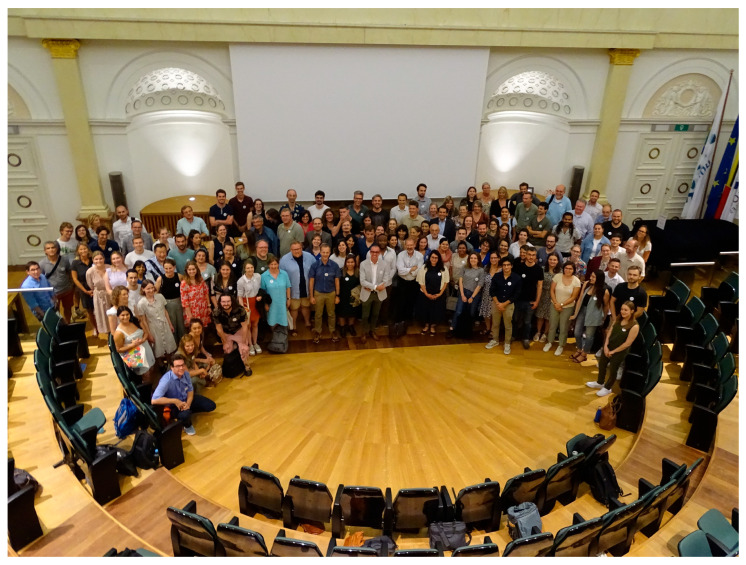
Group picture of participants of the 2nd Belgian Society for Viruses of Microbes (BSVoM) symposium in the Academic Room of the University of Liège, Belgium (141 participants from 10 countries).

The Belgian Society for Viruses of Microbes (BSVoM) organized its second symposium on 8 September 2023 in the Academic Room of the University of Liège. The meeting program covered three thematic sessions launched by international keynote speakers (Figure 1). Prof. Bas Dutilh (University of Jena, Jena, Germany) and Prof. Daniel Nelson (University of Maryland, College Park, MD, USA) presented during the first two sessions dedicated to “Fundamental research in phage ecology and biology”. The third session, focusing on “Present and future applications of phages”, was introduced by two additional keynote lectures given by Prof. Lone Brøndsted (University of Copenhagen, Copenhagen, Denmark) and Prof. Patrick Soentjens (Queen Astrid Military Hospital, Brussels/Institute of Tropical Medicine, Antwerp, Belgium). Nine selected talks were given along the day and a pitch session was organized with eight student pitches selected by the scientific committee prior to the poster session which included thirty posters (Table 1). The meeting hosted 141 participants (Figure 2) from 10 countries working in academic research (from Belgium: ULB, VUB, UGent, KU Leuven, UNamur, ULiège, UCLouvain), for a federal agency or research institute (Sciensano, ILVO), in hospitals (Queen Astrid Military Hospital, CHULiège) or in companies and foreign research centers (e.g., Luxembourg Institute of Science and Technology, Erasmus University Medical Center, University Medical Center Utrecht).

## 2. Scientific Sessions

The BSVoM symposium started with the session on fundamental research in phage ecology and biology. It was composed of two parts, with the first one chaired by Prof. Jelle Matthijnssens (KU Leuven). The keynote speaker of this session was Prof. Bas Dutilh from University of Jena in Germany. Prof. Dutilh and his colleagues are investigating the Microverse, as the most complex system known to mankind, using high-throughput experiments and omics data of various flavors, combined with innovative computational analyses. To understand microbiomes in their context, Dutilh’s team builds computational models of the various processes, which drive microbial functioning and dynamics [1,2,3,4,5,6]. For his work, Prof. Dutilh has received prestigious awards, including NWO Veni/Vidi, ERC Consolidator, and Alexander von Humboldt Professorship. During his talk, the focus was on characterization of generalist versus specialist microbes. The term “social niche breadth” (SNB) was introduced, which is a quantitative measure of the niche, where environment of the microorganisms is defined by its community. Generalists, as fast-growing opportunists, often locally outcompete specialists and are variable across communities [6]. The genome size of generalists is not larger than that of specialists. However, genomes vary between generalists in terms of sizes and content. Genomes of specialists belonging to one genus are similar. Generalists were found to be older than specialists. Prof. Dutilh announced also “ICTV Taxonomy Challenge”, challenging all bioinformaticians to automate viral classification.

The second speaker, Ella Sieradzki from the “Ecole Centrale de Lyon”, described phage dynamics in soil ecosystems [7,8]. Using isotope incorporation into viral and microbial DNA, phage–host temporal dynamics were characterized, revealing taxon-specific trends in viral–host dynamics. Recent hypotheses suggest predomination of temperate phages in soil. Ella Sieradzki showed that wet-up of the soil is dominated by phages in lytic cycles and that phages account for up to 46% of bacterial death one week after wet-up. This contributes to turnover of soil microbial biomass and CO_2_ efflux after wet-up of seasonally dry soils. 

The next speaker, Lore Van Espen (KU Leuven), presented her research on phage populations in more than 1000 fecal samples in terms of taxonomic classification, lifestyle and bacterial host. The group utilized high-quality metagenome-assembled genomes (MAGs) from the same samples. According to their analysis, 69% of the phages were predicted to be virulent. Prophages from unexpected groups (Crassphages, Inoviruses and Microviruses) were also detected. About 5% of the phages were predicted to possess a broad host range, since they could be linked to multiple bacterial hosts belonging to different genera, families, or even phyla. Hundreds of potentially novel phage genera were discovered. Their research may help to understand the potential role of phages in human health.

This session was concluded with a talk given by the invited speaker Prof. Daniel Nelson (University of Maryland, College Park, MD, USA). He explained the structure of the *Escherichia coli* O157:H7 bacteriophage CBA120 tailspike complex. Kuttervirus phages are equipped with up to four tailspike proteins (TSP1-4), which are responsible for degradation of bacterial host lipopolysaccharides [9,10,11,12]. CBA120 infects *E. coli* O157:H7 but also *E. coli* O77, O78, and *Salmonella* enterica serovar Minnesota, thanks to its different TSPs. TSPs are multidomain trimers with domains responsible for enzymatic cleavage and additional domains facilitating TSP assembly. Nelson’s group used mutagenesis and analytical ultracentrifugation techniques to map the interactions of CBA120 TSPs forming a branched complex.

The second part of the symposium, also on the topic of Fundamental research in ecology and phage biology, was chaired by Prof. Gipsi Lima-Mendez from the University of Namur, Belgium. The session started with the talk from Susanna Grigson (Flinders University, Adelaide, SA, Australia) about Phynteny: Synteny-based annotation of viral genes. Susanna pointed out that efficient annotation is fundamental to unraveling many aspects of viral processes [13,14]. Commonly used methods result in the assignment of the biological function to only 35% of the phage genes. While traditional methods are sequence homology-based, a novel freely accessible bioinformatic tool, called Phynteny, employs gene order. A long short-term memory model was trained on around one million viral sequences. Phynteny provides high-quality annotation for up to 64% of the phage genes.

The next speaker, Ekaterina Jalomo-Khayrova (Philipps-Universität Marburg, Marburg, Germany) talked about the molecular machinery evolved by prophages to control the lytic or lysogenic cycle. Master repressor MrpR of the heat-sensitive prophage SPβ c2 mutant, present in the genome of *Bacillus subtilis*, was analyzed as a decision maker [15,16,17]. A single nucleotide exchange in the *mrpR* gene was found responsible for the temperature sensitivity of the protein. MrpR was characterized as a DNA-binding protein, which lost recombinase activity and evolved into a repressor protein. As a result of binding of MrpR to several repeated elements in intergenic regions of the prophage genome, downstream gene expression is repressed and lysogeny is maintained.

The following talk by Albinas Cepauskas (ULB) covered the innate immune system of bacteria. Albinas explained that bacterial immunity, similar to the eukaryotic immune system, requires phage-specific triggers that are pathogen-associated molecular patterns-like (PAMPs-like). He presented genetic, biochemical and structural analyses of CapRelSJ46—a fused toxin-antitoxin (TA) system, present in *E. coli* [18]. While the N-terminus of CapRelSJ46 acts on tRNAs, leading to translation inhibition, the C-terminus blocks the pyrophosphorylating activity of the N-terminal part. Additionally, the C-terminus binds to phage major capsid proteins, which were freshly produced, serving as a highly specific sensor. This leads to the release of the auto-inhibition of the N-terminus and consequently abortive infection.

The final talk of this session was given by Enrico Tatti from Biolog, one of the sponsors of the conference. Biolog provides relevant solutions for functional phenotypic metabolic characterization and identification of microorganisms. Their OmniLog system has already been used in phage therapy related research [19,20]. Recently they have developed Odin, which apart from the measurement of cell respiration based on redox-sensitive dye reduction data (as in the OmniLog system), also measures bacterial growth based on turbidity.

Similar to the inaugural BSVoM conference, a pitch session was organized before the launch of the poster session, chaired by Prof. Gipsi Lima-Mendez (UNamur). This offers early career researchers the opportunity to shed light on their poster in three minutes (Table 1). Eight pitches were selected by the scientific committee. Laura Bessems (UZ Leuven) spoke about the optimization of phage therapy for difficult-to-treat musculoskeletal infections. Salomé Desmecht (ULiège) shared her first results about the isolation, in vitro characterization and efficacy assessment in *Galleria mellonella* larvae of four phages targeting *Aeromonas salmonicida*. Lene Bens (KU Leuven) exposed a challenging opportunity for phage therapy to treat hidradenitis suppurativa [21]. Céline Antoine (ULiège) talked about the genomic analysis and in vitro/in vivo characterization of phage-resistant *E. coli* K1 isolates. Wouter Magnus (VUB) presented the transcription regulatory program of the ssv1 virus infecting the thermoacidophilic archaeon *Saccharolobus solfataricus*. Claudia Campobasso (KU Leuven) discussed the potential key role of the baseplate protein of a *Staphylococcus aureus* phage with antibiofilm activity. Diana Bittremieux (ULB and UNamur) presented her research on the discovery of new antiviral mechanisms in oceanic bacteria using ADAM. Fanny Laforêt (ULiège) closed this pitch session by presenting the impact assessment of vB_KpnP_K1-ULIP33 phage on the human gut microbiota using a dynamic in vitro model [22]. The best pitch talk award went to Lene Bens from KU Leuven.

The second session of the conference was chaired by Dr. Pieter-Jan Ceyssens (Sciensano) and addressed the topic of present and future applications of phages. This session began with two keynote lectures presented by Prof. Lone Brøndsted from the University of Copenhagen, Copenhagen, Denmark, and Prof. Patrick Soentjens, head of the Centre for Infectious Diseases of the Queen Astrid Military Hospital in Brussels and chief physician at the Institute of Tropical Medicine in Antwerp, both in Belgium. Prof. Brøndsted and her team are currently focusing on in-depth analysis of phage biology, phage–host interactions and exploiting the antimicrobial potential of phages and phage proteins for novel approaches to combat pathogenic bacteria. In her keynote lecture, Prof. Brøndsted presented a collection of new phages infecting commensal antibiotic-resistant *E. coli* from livestock that pose risks to humans by transferring resistances to pathogenic strains. She discussed the different strategies of these phages to adjust to various host receptors, while maintaining specificity, and their potential use for animal decolonization was explored [23,24]. Moreover, her talk covered other examples of phage–host interactions in *E. coli*, contributing to our understanding of such interactions in complex environments and their societal benefits. Prof. Soentjens shared his vision on establishing a phage therapy hub in Belgium, based on the Belgian experience in the field. He first highlighted a clinical case involving the successful rescue of a 15-month-old child using a combination of phage and antibiotic therapy at “Hôpital Saint-Luc” in Brussels in 2018, as documented in Van Nieuwenhuyse et al., 2022 [25]. Prof. Soentjens then discussed the various elements and stakeholders that enable phage therapy in Belgium, including the magistral phage framework introduced in 2018 and the clinical coordination platform for phage therapy, thanks to which over 100 patients have already benefited from phage treatment [26].

The session continued with a presentation by Eveline-Marie Lammens from KU Leuven focusing on synthetic biology. Her research involves characterizing genetic parts of phage origin for implementation in *Pseudomonas* species such as *P. putida* to use them as versatile production hosts for synthetic biology applications. In particular, T7-like *Pseudomonas* phages were explored as potential sources of genetic parts given their co-evolution with bacterial hosts and their ability to manipulate host cells. Analysis of their genomes resulted in the identification of potent phage terminators and optimized T7-like RNA polymerases, their corresponding promoters and lysozymes. This work resulted in a tailored T7 expression system for *P. putida* and *P. aeruginosa*, showcasing the value of phages as sources of synthetic biology components [27].

Then, Roberto Vázquez from Ghent University took the floor to tackle the topic of phage lysins and their antibacterial potential against multidrug-resistant bacteria. Although presenting a broader spectrum compared to phages, their specificity determinants are still poorly understood. Dr. Vázquez systematically analyzed nearly 200 staphylococcal endolysins, focusing on their cell wall-binding domains (CBDs) using cutting-edge techniques. This study revealed an evolutionary convergence in staphylococcal lysins towards a trimodular structure bearing CBDs showing the SH3 fold in three different variants. Despite this convergence, there was remarkable variability in binding profiles, suggesting functional plasticity in these CBDs due to small structural adjustments in the SH3 fold. Additionally, he identified three critical amino acid residues in related CBDs that could contribute to their differential binding, offering insights for translational research on endolysins. 

Following these biotechnological aspects, the session shifted to a medical topic, highlighting an example of phage therapy applied in the context of burn wound infections, specifically those caused by *Staphylococcus aureus*, a major contributor to these life-threatening infections. Michele Molendijk from the Erasmus Medical Center in Rotterdam, Netherlands, presented an ex vivo model using surplus human skin to assess the effectiveness of phage therapy for burn wounds infected with a methicillin-resistant *S. aureus* strain. The study initially revealed that single treatments with either phages or antibiotics did not significantly reduce bacterial counts after 24 h. However, more notably, Michele Molendijk’s findings demonstrated that multiple phage treatments, particularly at higher doses, and pre-treatment of the skin with phages each led to a significant decrease in bacterial counts. These results are promising for burn victim management, as it is crucial to remember that the global mortality rate associated with bacterial infections in such cases remains high.

Manon Nuytten from UCLouvain then discussed the use of phages as a diagnostic tool, here for the development of Lateral Flow Assays (LFAs) based on phage proteins. LFAs, widely employed in point-of-care testing (e.g., COVID-19 self-test), are crucial in various fields but face limitations with traditional antibodies, such as variability in specificity and stability, and high costs. Phage proteins, involved in processes like adsorption and lysis, can serve as alternative bioreceptors for LFAs, but the understanding of their interface interactions is lacking. The emergence of precise bioinformatics tools such as AlphaFold2 has significantly facilitated the microstructural characterization and engineering of phage proteins, ultimately improving the performance of phage-based biosensors. In her talk, Manon Nuytten focused on the *Bacillus cereus* group which includes pathogenic species, emphasizing the importance of their rapid detection in the agro-food sector to prevent food poisoning. Through in silico analysis of phage affinity proteins targeting *B. cereus* used in an LFA format, the study identified regions involved both in the binding to LFA interfaces and bacterial cells, contributing to the optimization of protein performance in these tests.

The final presentation of the conference was delivered by Johan Quintens from Vésale Biosciences, a Belgian biotech established in 2018, focusing on personalized phage-based therapies. He outlined the four key pillars that underpin their efforts to offer successful phage therapy in the near future. The first pillar involves the development of a phagogram (Luminophage^®^) to determine the targeted bacteria’s sensitivity to a collection of phages, facilitating the selection of the most suitable phage. The second key point emphasized by Johan Quintens is the importance of maintaining a library of active phages against pathogens present in Western European hospitals, particularly targeting multidrug-resistant strains of *E. coli*, *S. aureus*, *K. pneumoniae* and *P. aeruginosa*. To achieve this, various networks of European and international partners have been established to ensure a comprehensive collection of phages and multidrug-resistant bacterial strains. The third pillar discussed relevant aspects of phage production, which must adhere to good manufacturing practice (GMP) standards, while maintaining flexibility. An efficient and integrated supply chain is essential to deliver standardized phages to hospital pharmacies, representing the final step in this approach. Through this innovative platform, Vésale Biosciences aims to address a patient’s specific bacterial infection with high precision and efficiency.

## 3. Conclusions

This second symposium was a success both in terms of the scientific quality of the presentations and the networking possibilities with stimulating exchanges between researchers, regulatory stakeholders and companies. The president of the Belgian Society for Viruses of Microbes, Prof. Yves Briers, took advantage of this symposium to launch the Phage Valley concept that will put the spotlight on the exceptionally high density of researchers investigating viruses of microbes along with the successful triple helix approach between academia, industry and government in Belgium.

## Figures and Tables

**Table 1:** Posters presented during the 2nd BSVoM ^1^ symposium.

Authors	Poster Title
Fundamental research in phage ecology and biology	
Antoine C., Laforêt F., Blasdel B., Fall A., Duprez J.N., Delcenserie V. ^2^ Thiry D. ^2^	Genomic analysis and in vitro/in vivo characterization of phage resistant *E. coli* K1 isolates
Bittremieux D., Dugauquier R., Kurata T., Abdullah M. Pelzer L., Garcia Pino A., Hauryliuk V., Lima Mendez G.	Discovery of mechanisms of interactions between bacteria and bacteriophages in the human gut microbiome
Culot A., Abriat G.	Enhancing phage therapy safety: reliable and sensitive phage genome annotation with rTOOLS2 high-throughput pipeline
Dugauquier R., Bittremieux D., Abdullah M., Shyrokova O., Kurata T., Roomans C., Garcia-Pino A., Hauryliuk V., Lima-Mendez G.	Discovery of new antiviral mechanisms in oceanic bacteria using ADAM
Egido J., Dekker S., Rooijakkers S., Bardoel B., Haas P.J.	Bacteriophages against *Pseudomonas aeruginosa* are inhibited by complement in human serum
Faye L., Sijmons S., Matthijnssens J.	Optimization of virome enrichment method for human respiratory samples
Latka A., Dams D., Squeqlia F., Scholiers L., Otwinowska A., Olejniczak S., Maciejewska B., Gutiérrez D., Berisio R., Drulis-Kawa Z., Briers Y.	Understanding the RBP modules function of KP32 as a podophage model
Lechuga A. ^2^, Lood C. ^2^, Aerts Y., Macián A., Kyrkou I., Lavigne R.	Isolation and characterization of spontaneously induced *Pseudomonas* temperate phages
Longin H., De Vrieze L., Beahan B., Hendrix H., Lavigne R., van Noort V.	Viral acetyltransferases: a novel, yet widespread host-hijacking strategy in *Pseudomonas* phages
Magnus W. ^3^, Boon M., Stedman K., Lavigne R., Peeters E.	Transcription regulatory program of the SSV1 virus infecting the thermoacidophilic archaeon *Saccharolobus solfataricus*
Shaikh H.M., Brussaard C.P.D., De Keulenaer S., Van Nieuwerburgh F., Piedade G.J., De Rijcke M.	Comparative assessment reveals significant output differences between VIPCAL and linear regression in viral reduction assays
Steinmetz J., Schiavolin L., Smeesters P., Botteaux A.	Analysis of Group A *Streptococcus* lytic phage interactions with human serum
Present and future applications of phages	
Bens L. ^3^, Almeida M., Green S., Wagemans J., Lavigne R.	Hidradenitis suppurativa: A challenging opportunity for phage therapy
Bessems L., Chen B., Uyttebroek S., Devolder D., Lood C., De Munter P., Debaveye Y., Depypere M., Spriet I., Van Gerven L., Wagemans J., van Noort V., Lavigne R., Metsemakers W.J.	Optimization of bacteriophage therapy for difficult-to-treat musculoskeletal infections: from bench to bedside and vice versa
Campobasso C., Wagemans J., Di Luca M., Lavigne R.	Antibiofilm activity of a *Staphylococcus aureus* phage: potential key role of its baseplate protein
De Maesschalck V., Gutiérrez D., Paeshuyse J., Briers Y., Lavigne R.	Burnzymes as anti-inflammatory, third-generation lysins targeting burns infected with *Acinetobacter baumannii*
Desmecht S., Antoine C., Laforêt F., Touzain F., Vermeersch M., Leroux A., Duprez J.N., Schonbrodt A., Lieffrig F., Thiry D.	Isolation, in vitro characterization and efficacy assessment in *Galleria mellonella* larvae of four bacteriophages targeting *Aeromonas salmonicida*
Diderich J., Antoine C., Laforêt F., Desmecht S., Duprez J.-N., Miyamoto T., Thiry D.	Stability and lytic activity assessment of bacteriophages targeting *Staphylococcus aureus* causing bovine mastitis in milk
Fortuna K.J., Szoboszlay M., Holtappels D., Lavigne R., Tebbe C.C., Wagemans J.	Assessing the environmental biosafety of phage-based biocontrol applications
Goossens M. ^3^, Glonti T., Dams D., Łątka A., Pirnay J.-P., Briers Y.	Towards an alternative approach for personalized phage therapy: Instant and on-site production of SynPhages (Synthetic Phages)
Laforêt F. ^3^, Antoine C., Lebrun S., Gonza I., Goya-Jorge E., Douny C., Duprez J.-N., Scippo M.-L., Taminiau B., Daube G., Fall A., Thiry D. ^2^, Delcenserie V. ^2^	Impact assessment of vB_KpnP_K1-ULIP33 bacteriophage on the human gut microbiota using a dynamic in vitro model
León-Quezada R., Zampara A., Vázquez R., Moral-Lopez P., Briers Y., Brøndsted L.	INNOLYSINS: Novel antibacterials against *Salmonella*
Leonard C., Desmecht S., Antoine C., Laforêt F., Duprez J.N., Piedfort O., Fontaine J. ^2^, Thiry D. ^2^	Isolation and characterization of five lytic bacteriophages against *Pseudomonas aeruginosa* causing canine otitis externa
Navez M., Antoine C., Laforêt F., Taminiau B., Delcenserie V., Thiry D.	In vitro effect on piglet gut microbiota and in vivo assessment of newly isolated bacteriophages against F18 Enterotoxigenic *Escherichia coli* (ETEC)
Pottie I., Duyvejonck L., Briers Y.	Breaking down walls: Towards a functional-based metagenomic discovery platform for phage-derived lysins
Tahzima R., Haegeman A., Simankov N., Soyeurt H., Massart S., De Jonghe K.	Unearthing the global soil microbial-associated virome and (u)MAPping the tectonics of its alphaFOLD2-aware structural diversity upon a One Health framework
Uyttebroek S., Dupont L., Wagemans J., Lavigne R., Coenye T., Van Gerven L.	In vitro efficacy of bacteriophages in the treatment of chronic rhinosinusitis-related biofilms
Vander Elst N., Bellemans J., Lavigne R., Briers Y., Meyer E.	A VersaTile-engineered endolysin improves early cloxacillin treatment in a preclinical mouse model for *Streptococcus uberis* mastitis: A proof-of-concept study
Vermeulen S., Nuytten A., Van Holle S., Van Mechelen E., Decostere B., Verhelst R., Dryoel J., Hulaj B., Otálora Rodriguez P., Van der heyden C.	PCR for the typing of the most common filamentous bacteria in wastewater treatment plants for targeted bacteriophage use
Willocx M., de Jode M., Laurent F., Merabishvili M., Lavigne R. ^2^, Ceyssens P.-J. ^2^, Vanhee C. ^2^	Pyrogen testing of phage therapeutic products

^1^ Belgian Society for Viruses of Microbes. ^2^ These authors contributed equally to this work. ^3^ Best poster and poster pitch award winners.

## Data Availability

Data are contained within the article.
